# Rapid and Accurate Detection of SARS Coronavirus 2 by Nanopore Amplicon Sequencing

**DOI:** 10.3389/fmicb.2022.735363

**Published:** 2022-04-06

**Authors:** Xiao-xiao Li, Chao Li, Peng-cheng Du, Shao-yun Li, Le Yu, Zhi-qiang Zhao, Ting-ting Liu, Cong-kai Zhang, Sen-chao Zhang, Yu Zhuang, Chao-ran Dong, Qing-gang Ge

**Affiliations:** ^1^Department of Pharmacy, Department of Intensive Care Unit, and Department of Medical Affairs, Peking University Third Hospital, Beijing, China; ^2^Institute of Materia Medica, Chinese Academy of Medical Sciences and Peking Union Medical College, Beijing, China; ^3^Beijing YuanShengKangTai (ProtoDNA) Genetech Co. Ltd., Beijing, China

**Keywords:** severe acute respiratory syndrome coronavirus 2, nanopore amplicon sequencing, RT-qPCR, viral detection, genome coverage

## Abstract

**Objective:**

We aimed to evaluate the performance of nanopore amplicon sequencing detection for severe acute respiratory syndrome coronavirus 2 (SARS-CoV-2) in clinical samples.

**Method:**

We carried out a single-center, prospective cohort study in a Wuhan hospital and collected a total of 86 clinical samples, including 54 pharyngeal swabs, 31 sputum samples, and 1 fecal sample, from 86 patients with coronavirus disease 2019 (COVID-19) from Feb 20 to May 15, 2020. We performed parallel detection with nanopore-based genome amplification and sequencing (NAS) on the Oxford Nanopore Technologies (ONT) minION platform and routine reverse transcription quantitative polymerase chain reaction (RT-qPCR). In addition, 27 negative control samples were detected using the two methods. The sensitivity and specificity of NAS were evaluated and compared with those of RT-qPCR.

**Results:**

The viral read number and reference genome coverage were both significantly different between the two groups of samples, and the latter was a better indicator for SARS-CoV-2 detection. Based on the reference genome coverage, NAS revealed both high sensitivity (96.5%) and specificity (100%) compared with RT-qPCR (80.2 and 96.3%, respectively), although the samples had been stored for half a year before the detection. The total time cost was less than 15 h, which was acceptable compared with that of RT-qPCR (∼2.5 h). In addition, the reference genome coverage of the viral reads was in line with the cycle threshold value of RT-qPCR, indicating that this number could also be used as an indicator of the viral load in a sample. The viral load in sputum might be related to the severity of the infection, particularly in patients within 4 weeks after onset of clinical manifestations, which could be used to evaluate the infection.

**Conclusion:**

Our results showed the high sensitivity and specificity of the NAS method for SARS-CoV-2 detection compared with RT-qPCR. The sequencing results were also used as an indicator of the viral load to display the viral dynamics during infection. This study proved the wide application prospect of nanopore sequencing detection for SARS-CoV-2 and may more knowledge about the clinical characteristics of COVID-19.

## Highlights

- Reference genome coverage was a prior indicator in severe acute respiratory syndrome coronavirus 2 (SARS-CoV-2) detection applying nanopore amplicon sequencing (NAS) compared with viral read number.
- Based on the reference genome coverage, a higher sensitivity and specificity of NAS in detecting SARS-CoV-2 was revealed through its comparison with reverse transcription quantitative polymerase chain reaction (RT-qPCR).
- Some long-term stored samples with negative RT-qPCR results tested positive using the NAS approach.
- Sequencing results using the NAS approach revealed that dynamic variations in both pharyngeal swabs and sputum samples had potential to evaluate disease progression, especially for patients diagnosed as coronavirus disease 2019 within 4 weeks after the first clinical manifestations exhibited.

## Introduction

The sudden emergence of the coronavirus disease 2019 (COVID-19) pandemic, caused by severe acute respiratory syndrome coronavirus 2 (SARS-CoV-2), has rapidly spread globally (Pollard et al., 2020; Zhu et al., 2020). Strengthening the national and regional laboratory diagnostic capacity for COVID-19 was one of the key measures to counter this global public health crisis (Tang Y. W. et al., 2020). Specifically, a rapid and accurate nucleic acid test method for SARS-CoV-2 detection and COVID-19 diagnostics is critical for disease control and patient treatment.

The COVID-19 outbreak was followed by the characterization of the whole viral genome, which helped to develop several molecular diagnostic methods (Tang X. et al., 2020). On February 4, 2020, real-time reverse transcription polymerase chain reaction (RT-PCR) assay for COVID-19 was authorized by the Centers for Disease Control and Prevention (FDA, 2020). Till now, the RT-PCR is considered as the most conclusive molecular diagnostic approach. In addition, the cycle threshold (*C*_t_) value of reverse transcription quantitative polymerase chain reaction (RT-qPCR) is also used as an indicator of the viral load in samples to estimate the viral dynamics in different periods or body sites during the infection. However, it suffers from high false-negative rates due to variations in the viral ribonucleic acid (RNA) sequences in the *ORF1ab* and *N* genes, which the primers target, raising the call for more sensitive methods (Wang Y. et al., 2020).

Next-generation sequencing (NGS) has been used for SARS-CoV-2 detection and research as early as its emergence. However, the higher costs, longer detection periods, and complicated operating steps have limited its application, even that several improved protocols based on library capture or viral genome amplification have been proposed (Chen et al., 2020; Nasir et al., 2020; Xiao et al., 2020). Oxford nanopore sequencing is a promising new generation of sequencing technology that features real-time data generation and long read length (Gu et al., 2021). Therefore, SARS-CoV-2 detection combining viral genome amplification and nanopore sequencing provides comprehensive genome coverage and lower time cost (Wang M. et al., 2020). Our team reported a patient finally confirmed as a COVID-19 case using nanopore-based genome amplification and sequencing (NAS), while the routine RT-qPCR was negative due to a viral mutation within the primer region (Mehta et al., 2020). Furthermore, this method allows a more convenient and efficient SAR-CoV-2 genomic surveillance, helping to identify mutations that have an impact on virulence and transmissibility (Bull et al., 2020; Lu et al., 2020). However, the performance of this approach has not been widely evaluated, particularly using clinical samples and in comparison with RT-qPCR.

We obtained NAS data from a cohort including hospitalized COVID-19 patients with mild (or moderate) or severe (or critical) disease and healthy controls to gain insight into the identification of SARS-CoV-2-specific genome compared with RT-qPCR, which may further serve as a better method for SARS-CoV-2 detection and an indicator of the viral load dynamics during infection.

## Materials and Methods

### Specimen Collection

We carried out a single-center, prospective cohort study in a Wuhan pulmonary hospital. Eighty-six patients with a minimum age of 18 years and were confirmed as having COVID-19 were consecutively enrolled from Feb 20 to May 15, 2020. On the other hand, 27 patients who tested negative for COVID-19 were recruited into the control group. Patients were diagnosed with COVID-19 according to the *Diagnosis and Treatment Protocol of COVID-19* (the 6th–8th tentative version^1^) issued by the National Health Commission of the People’s Republic of China; if they had any epidemiological history plus any two clinical manifestations (fever and/or respiratory symptoms; the aforementioned imaging characteristics of COVID-19; normal or decreased white blood cell count, and normal or decreased lymphocyte count in the early stage of onset) or all three clinical manifestations if there was no clear epidemiological history; and if the etiological evidence was confirmed as a *positive* result for new coronavirus nucleic acid detected using RT-qPCR (*positive* was defined as *C*_t_ ≤ 35 according to the protocol offered by the clinical laboratory center). Patients were then classified into two groups: mild (or moderate) (fever, respiratory symptoms, etc., with or without abnormal lung imaging) and severe (or critical) (respiratory rate ≥ 30 per min, oxygen saturation ≤ 93% on room air at rest, arterial oxygen pressure/fraction of inspiration oxygen ≤ 300 mmHg, or progressive clinical symptoms and lung imaging showing >50% significant progression of the lesion within 24–48 h; even cause an aggravation of respiratory failure requiring mechanical ventilation, shock, or combined with other organ failure and requiring intensive care unit supervision). In addition to discarded pharyngeal swab, 5 ml discarded sputum sample or discarded fecal sample was collected from each patient during hospitalization and stored at −80°C after nucleic acid isolation. The demographic data were recorded. All of the clinical specimens and demographic data were obtained in accordance with the study protocol, which was approved by the Ethical Review Board of Peking University Third Hospital in March 2020, with informed consent from participants being exempted (IRB00006761-M2020083).

### Ribonucleic Acid Extraction and Routine Reverse Transcription Quantitative Polymerase Chain Reaction Detection of Severe Acute Respiratory Syndrome Coronavirus 2

We performed parallel detections, one applying RT-qPCR and the other one applying SARS-CoV-2 genome amplification and sequencing using the Oxford Nanopore Technologies (ONT) platform based on the ARTIC protocol^2^. Firstly, total RNA was extracted from 200 μl specimen with an automatic nucleic acid extractor (EXM3000; Zybio Inc., Chongqing, China) according to the manufacturer’s instructions. A 60-μl elution volume was obtained for each sample in total after three extractions in parallel. Five microlters total RNA was used for real-time RT-qPCR, which targeted the *ORF1ab* and *N* genes using the Novel Coronavirus (2019-nCoV) nucleic acid detection kit (ZC-HX-201-2; BioGerm, Shanghai, China). RNase P was used as the endogenous internal standard. Real-time RT-qPCR was performed in the following conditions: 50°C for 10 min and 95°C for 5 min, then 40 cycles of amplification at 95°C for 10 s and 55°C for 40 s with the 7,500 Real-Time PCR System (Applied Biosystems, Bedford, MA, United States). The criteria for evaluation of the results were as follows: *C*_t_ value ≤ 38 as positive and simultaneous positive determination of both *ORF1ab* and *NP* genes.

### Severe Acute Respiratory Syndrome Coronavirus 2 Detection by Nanopore Amplicon Sequencing

During SARS-CoV-2 genome amplification and library construction, 16 μl total RNA was used as a template for reverse transcription with the LunaScript RT SuperMix kit [E3010L; New England Biolabs (NEB), Ipswich, MA, United States] according to the manufacturer’s instructions. A 5-μl cDNA production was then used as a template for targeted amplification of the SARS-CoV-2 genome with the Q5 Hot-Start High-Fidelity 2x Master Mix (M0494; NEB, Ipswich, MA, United States) and primer pools A and B (PCR tiling of COVID-19 virus, version 4; ONT, Didcot, United Kingdom). PCR was performed in the following conditions: 98°C for 30 s, followed by 30 cycles of amplification at 98°C for 15 s and 65°C for 2 min with the Veriti DX 96-well Thermal Cycler (Applied Biosystems, Bedford, MA, United States). DNAse/RNAse-free water was assayed in each batch as a negative control. A synthetic double-strand SARS-CoV-2 sequence with a length of 820 bp (NC_045512.2: nt 13,005–13,824) was used as a positive control. PCR products were cleaned up with the same volume of Agencourt AMPure XP beads (A63881; Beckman, Brea, CA, United States). In the next stage, the Ultra II End-Repair/dA-Tailing Module (E7546; NEB, Ipswich, MA, United States) was used for end-prep reaction and the Next Ultra II Ligation Module (E7595; NEB, Ipswich, MA, United States) used for barcode ligation with Native Barcoding Expansion 96 (NBD-196; ONT, Didcot, United Kingdom). MinION library preparation was performed according to the manufacturer’s instructions in the Ligation Sequencing Kit (SQK-LSK109; ONT, Didcot, United Kingdom). Different barcoded samples were pooled with equal masses. The concentration of each library was measured using Qubit 4.0 Flurometer (Invitrogen, Carlsbad, CA, United States), and the volume of each library was then calculated to make an equimolar pool of libraries. The pool of libraries was finally sequenced using the ONT MinION platform (MinKNOW 19.12.5).

### Sequencing Data Analysis

After Nanopore sequencing, the raw data were filtered using NanoFilt software^3^. High-quality reads (*Q* ≥ 10) were retained. The clean data were mapped to the reference genome of SARS-CoV-2 isolate Wuhan-Hu-1 (accession no. NC_045512.2) using Minimap2 software (Li, 2018). The reads that could be mapped to the reference with *a* ≥ 50 mapping quality score (MAPQ) were then compared with the reference using BLAST software (Altschul et al., 1990). When the alignment of a read with reference by BLAST covered >80% of the whole read and the sequence identity was ≥90%, this read was recognized as a SARS-CoV-2 read. For each sample, the sequencing depth of each position and the number of times that position was read were calculated with SAMtools mpileup. The calculation was based on the mapping results of the confirmed SARS-CoV-2 reads (Li et al., 2009). Genome coverage was determined using the proportion of sequenced genome position. It was calculated based on the number of sequenced reference position, which was divided by the reference genome size (29,903 bp).

### Statistical Analysis

To evaluate the detection ability of NAS for SARS-CoV-2 in clinical samples, we calculated the sensitivity and specificity of both the sequencing and the RT-qPCR results. Samples collected from COVID-19 patients were considered positive, while the nasopharyngeal swabs from others were considered negative for the virus. The cutoff of the RT-qPCR outputs was followed by the instruction of 2019-nCoV nucleic acid detection kit used (*C*_t_ ≤ 38). The cutoff values of the read number and genome coverage from NAS were estimated by calculating the receiver operating characteristic (ROC) curve and the area under the curve (AUC) generated using the pROC package in R (Robin et al., 2011). Using different parameters of sequencing results, the sensitivity and specificity were then calculated once the maximum AUCs were obtained. Comparison of the *C*_t_ values and the genome coverage between the two groups was performed with a non-parametric comparison using the wilcox.test() function in R package. A *p*-value < 0.05 was considered statistically significant.

## Results

### Rapid Severe Acute Respiratory Syndrome Coronavirus 2 Whole-Genome Detection by the Nanopore Amplicon Sequencing Approach

**FIGURE 1:**
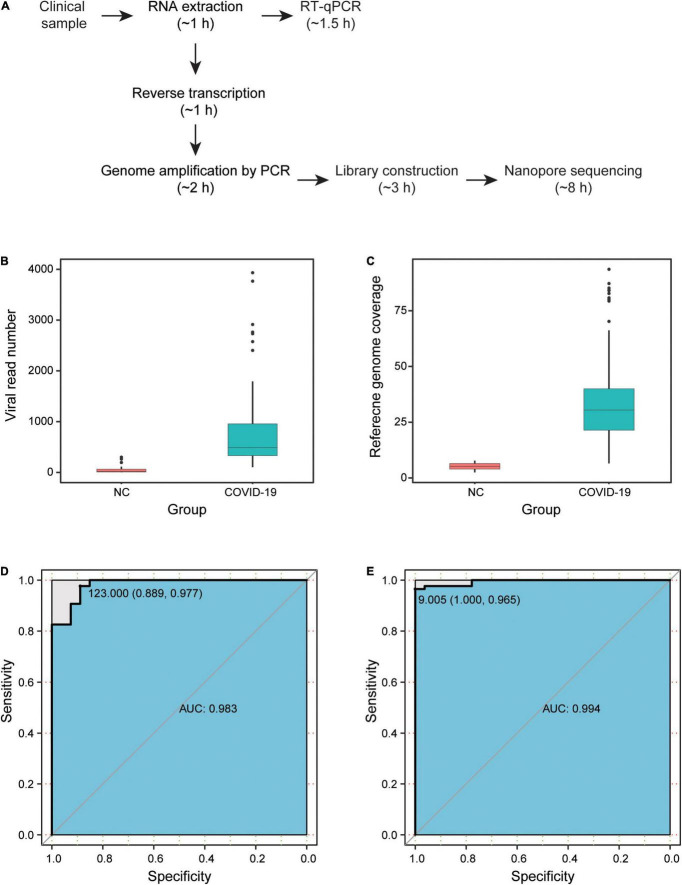
Results of severe acute respiratory syndrome coronavirus 2 (SARS-CoV-2) NAS. **(A)** Detection time of NAS and RT-qPCR. **(B)** Viral read number of samples in the COVID-19 and NC groups. **(C)** Reference genome coverage of samples in the COVID-19 and NC groups. **(D)** ROC curve based on the viral read number. **(E)** ROC curve based on the reference genome coverage. NAS, nanopore amplicon sequencing; RT-qPCR, reverse transcription quantitative polymerase chain reaction; ROC, receiver operating characteristic; NC, negative controls; COVID-19, coronavirus disease 2019.

We enrolled 86 hospitalized patients diagnosed with COVID-19, with a median age of 65 years [interquartile range (IQR) = 50–76 years], with 52.3% being men (45 patients). There were 86 clinical samples collected from these patients, including 54 pharyngeal swabs, 31 sputum samples, and 1 fecal sample. To test the SARS-CoV-2 detection performance, we carried out a parallel design based on the NAS and RT-qPCR approaches for 86 clinical samples from COVID-19 patients (COVID-19 group) and 27 negative controls (NC group). In total, the detection of all 113 samples was performed in three R9.4 chips, which was completed in 8 h (Figure 1A).

After filtering low-quality data and carrying out the two-step identification of reads from SARS-CoV-2, the median number of viral read was 585.5 (IQR = 384–2,755) in the 86 clinical samples. According to the ARTIC protocol, the overlapping amplicons covered nearly the whole viral genome, except for the 3′ and 5′ untranslated regions, and the median size of amplicons was 392 bp (IQR = 383–400). Among the high-quality clean data, the median length of nanopore reads was 495 bp (IQR = 346–532). Surprisingly, viral reads were also identified in all 27 samples of the NC group, with a median number of 20 (IQR = 8–64). Based on the mapping results of the viral reads to the reference genome of SARS-CoV-2 isolated from Wuhan-Hu-1, the median reference genome coverage was 30.5% (IQR = 21.4–40.0%) in the COVID-19 group and was 5.2% (IQR = 4.0–6.5%) in the NC group. Both the read number and the genome coverage of the virus for the COVID-19 group were significantly higher than those of the NC group (Figures 1B,C).

### High Sensitivity and Specificity of Nanopore Amplicon Sequencing

The sensitivity and specificity of NAS were then estimated by drawing the ROC curve and calculating the AUC based on the viral read number and reference genome coverage in the COVID-19 and NC groups (Figures 1D,F). Two samples from COVID-19 patients were determined as negative, with a read number cutoff of 123. Three NC samples were then determined as positive. Subsequently, we obtained the highest sensitivity of 97.7% (84/86), along with a specificity of 88.9% (24/27) and an AUC of 0.983 (95% CI = 0.9626–1). With the genome coverage cutoff set as 9.005%, three samples from COVID-19 patients were determined as negative, while none of the NC samples were determined as positive. The highest AUC of 0.994 (95% CI = 0.9862–1) was then obtained, along with a sensitivity of 96.5% (83/86) and a specificity of 100% (27/27). These results revealed the high sensitivity and specificity of NAS for the detection of SARS-CoV-2 (Table 1).

**TABLE 1:** Sensitivity and specificity of nanopore amplicon sequencing (NAS) and reverse transcription quantitative polymerase chain reaction (RT-qPCR).

	Group		Sensitivity (%)	Specificity (%)
	COVID-19	NC		
NAS viral read number				
≥123	84	3	97.7	88.9
<123	2	24		
NAS coverage				
≥9.005%	83	0	96.5	100
<9.005%	3	27		
RT-qPCR				
+	69	1	80.2	96.3
−	17	26		

*NAS, nanopore amplicon sequencing; RT-qPCR, reverse transcription quantitative polymerase chain reaction; NC, negative controls; COVID-19, coronavirus disease 2019; +, positive; −, negative.*

**FIGURE 2:**
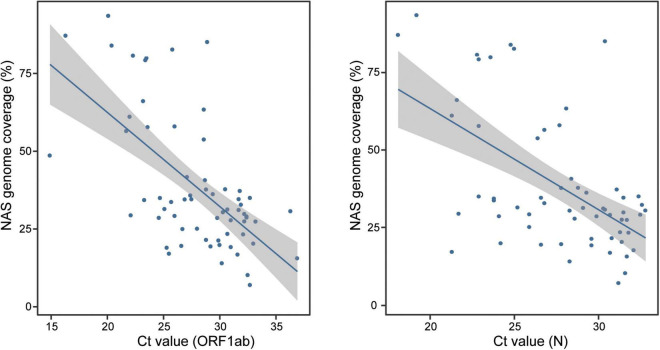
Consistency of the genome coverage of NAS and the *C*_t_ value of RT-qPCR detection of the *ORF1ab* and *N* genes. *NAS*, nanopore amplicon sequencing; *C*_t_, cycle threshold; *RT-qPCR*, reverse transcription quantitative polymerase chain reaction.

We next performed parallel RT-qPCR detection of SARS-CoV-2 in these samples to compare the efficiency of both two methods. With the standard *C*_t_ value cutoff set as 38, 17 samples from COVID-19 patients were negative and one NC sample was positive. A sensitivity of 80.2% (69/86) and a specificity of 96.3% (26/27) were obtained (Figure 1E). Compared with the performance of RT-qPCR, NAS showed obviously higher sensitivity and specificity. In addition, the genome coverage generated by the NAS method was in line with the *C*_t_ value of RT-qPCR, with an *R*^2^ of 0.382 with the *C*_t_ value of the *ORF1ab* gene amplification and an *R*^2^ of 0.311 with the *C*_t_ value of the *N* gene amplification (Figure 2).

### The Viral Load in Sputum Estimated by the Nanopore Amplicon Sequencing Approach Might Be Related With the Severity and Duration of Infection

The *C*_t_ value of RT-qPCR has been widely applied as a surrogate indicator for viral load. However, the sensitivity of RT-qPCR was markedly lower than that of NAS in the detection of the long-term stored samples in this study (80.2% *vs*. 96.5% as reference genome coverage). The reference genome coverage calculated from the NAS detection and the *C*_t_ value of RT-qPCR were highly consistent. We therefore estimated the viral load of the samples using both values (the *C*_t_ value of the *ORF1ab* gene was applied due to its higher consistency with the genome coverage generated by the NAS approach) and then compared their estimated efficiency, followed by analysis of the variations in the viral loads of COVID-19 patients during the period of infection.

**FIGURE 3:**
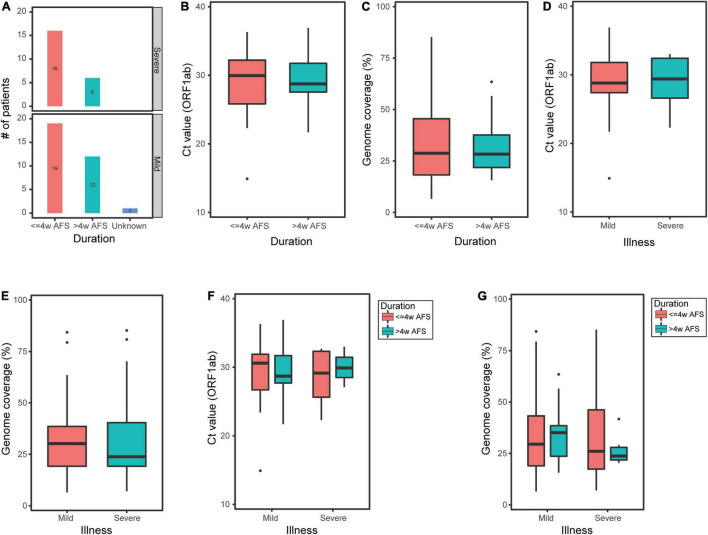
*C*_t_ value of *ORF1ab* and reference genome coverage in pharyngeal swabs from COVID-19 patients. **(A)** Number of pharyngeal swabs from mild or moderate patients (mild group) and critical or severe patients (severe group) within less or longer than 4 weeks after onset of clinical manifestations (≤*4w AFS* or >*4w AFS*, respectively). **(B,C)** *C*_t_ value and reference genome coverage in pharyngeal swabs from patients with ≤4w AFS or >4w AFS. **(D,E)** *C*_t_ value and reference genome coverage in pharyngeal swabs from mild or severe groups. **(F,G)** *C*_t_ value and reference genome coverage in pharyngeal swabs in four groups based on both the time of diagnosis and the severity of patients. COVID-19, coronavirus disease 2019; AFS, after first showing clinical manifestations; C_t_, cycle threshold.

Among the 54 pharyngeal swabs, 35 samples were from patients within less than 4 weeks after onset of clinical manifestations (hereinafter referred to as “≤4w AFS”) and 18 samples were from patients within longer than 4 weeks after the first clinical manifestations exhibited (hereinafter referred to as “>4w AFS”) (the confirmed time of a patient was missing; Figure 3A). For these two groups, the median *C*_t_ values were 29.95 and 28.75 and the median genome coverage values were 28.77% and 28.31%, respectively (Figures 3B,C). Neither the reference genome coverage nor the *C*_t_ value in pharyngeal swabs was significantly different between the two groups of patients (Figures 3B,C). Thirty-two pharyngeal swabs were from mild or moderate patients (mild group) and 22 samples were from severe or critical patients (severe group). In these two groups, the median *C*_t_ values were 28.8 and 29.4 and the median genome coverage were 30.2% and 23.8%, respectively (Figures 3D,E). There was also no significant difference in both *C*_t_ values and genome coverage in the pharyngeal swabs between the two groups. We then divided the 54 pharyngeal swabs into four groups based on both the onset of clinical manifestations and the severity of disease: ≤4w AFS and mild (19 samples), ≤4w AFS and severe (16), >4w AFS and mild (12), and >4w AFS and severe (6). For these four groups, the median *C*_t_ values were 30.6, 29.2, 28.7, and 29.9 (Figure 3F) and the median genome coverage values were 29.5%, 26.1%, 35.1%, and 23.7%, respectively (Figure 3G). No significant difference was observed between any two groups.

**FIGURE 4:**
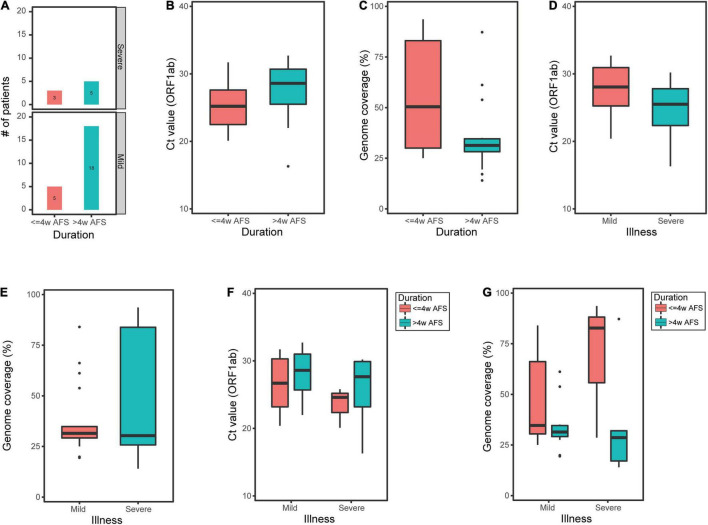
*C*_t_ value of *ORF1ab* and reference genome coverage in sputum samples from COVID-19 patients. **(A)** Number of sputum samples from mild or moderate patients (mild group) and critical or severe patients (severe group) within less or longer than 4 weeks after onset of clinical manifestations (≤*4w AFS* or >*4w AFS*, respectively). **(B,C)** *C*_t_ value and reference genome coverage in sputum samples from patients with ≤4w AFS or >4w AFS. **(D,E)** *C*_t_ value and reference genome coverage in sputum samples from mild or severe groups. **(F,G)** *C*_t_ value and reference genome coverage in sputum samples in four groups based on both the time of diagnosis and the severity of patients. *COVID-19*, coronavirus disease 2019; *AFS*, after first showing clinical manifestations; *C*_t_, cycle threshold.

In contrast, of the 31 sputum samples, 8 samples were from patients within ≤4w AFS and 23 samples were from patients within >4w AFS (Figure 4A). For these two groups, the median *C*_t_ values were 25.2 and 28.6 and the median genome coverage values were 50.4% and 31.3%, respectively (Figures 4B,C). The *C*_t_ values in the sputum were not significantly different between the two groups (Figure 4B); however, the median genome coverage values in the sputum from patients within ≤4w AFS were markedly higher than those from patients >4w AFS, but the difference was not significant (Figure 4C). Twenty-three sputum samples were from the mild group and 8 samples were from the severe group. For these two groups, the median *C*_t_ values were 28.1 and 25.5 and the median genome coverage values were 31.5% and 30.3%, respectively (Figures 4D,E). There was also no significant difference in both *C*_t_ values and genome coverage in the sputum between these two groups (Figures 4D,E). We then divided the 31 sputum samples into four groups based on both the time diagnosis and the severity of the disease: ≤4w AFS and mild (5 samples), ≤4w AFS and severe (3), >4w AFS and mild (18), and >4w AFS and severe (5). For these four groups, the median *C*_t_ values were 26.7, 24.6, 28.6, and 27.7 and the median genome coverage values were 34.7%, 82.8%, 31.4%, and 28.6%, respectively (Figures 4F,G). The genome coverage in ≤4w AFS and severe group was dramatically higher than those of the other groups, although the difference was not significant due to the limited number of samples, which was also in line with the feature of the *C*_t_ value (Figures 4F,G). Therefore, the viral load in sputum samples instead of that in pharyngeal swabs might be related to the severity of infection, particularly in patients with ≤4w AFS, which could be used as a secondary tool to evaluate the severity of SARS-CoV-2 infection.

## Discussion

In this study, we detected SARS-CoV-2 in 86 clinical samples from COVID-19 patients using the NAS method on the Nanopore minION sequencing platform. We calculated the viral read number and reference genome coverage in each sample to evaluate the detection results. We estimated the sensitivity and specificity of this method using these results and those from 27 negative control samples. Our analysis revealed both a higher sensitivity and a better specificity (96.5% and 100%, respectively) of NAS (based on genome coverage) compared with RT-qPCR (80.2% and 96.3%, respectively). The reference genome coverage of the viral reads was in line with the *C*_t_ value of RT-qPCR, which revealed that the reference genome coverage could also be used as an indicator of the viral load in samples. In addition, the total time cost was less than 15 h, which was also acceptable compared with that of RT-qPCR (∼2.5 h). These results showed that the NAS method was accurate and rapid for SARS-CoV-2 detection in clinical samples.

Our results revealed that the NAS detection approach used in this study would be more sensitive and accurate than that based on PCR alone. Firstly, dozens of positions along the viral genome were detected by overlapping amplification, whereas only one or two loci were detected by RT-qPCR. Secondly, the amplicons were sequenced and validated by subsequent sequence comparison. This whole viral genome detection led to a high sensitivity and a good specificity simultaneously. This approach was used to diagnose a COVID-19 patient, in which the routine RT-qPCR was negative due to a viral mutation on the primer site and only one read was obtained by NGS detection (Mehta et al., 2020).

The NAS approach might also be more valuable than those based on NGS for the detection of SARS-CoV-2. Numerous studies performed SARS-CoV-2 detection and research based on NGS and revealed great valuable knowledge about the genomic characteristics and transmission of the virus (Gudbjartsson et al., 2020; Lu et al., 2020; Rambaut et al., 2020). However, due to the fixed and prolonged run time of the NGS platform, the detection results are usually obtained in 2–3 days, which limited its application in clinical tests and rapid screening. In contrast, sequencing using the ONT minION platform is flexible due to its real-time nature. With the NAS method, we performed SARS-CoV-2 genome amplification firstly to enrich the viral nucleotide fragments. Therefore, the data required would be moderate in the subsequent sequencing, by which the detection time (15 h, or even shorter) would be reduced significantly compared to deep sequencing using the NGS platform. Meanwhile, a high accuracy was also achieved due to the subsequent data analysis based on the long sequencing read compared with NGS (400 bp for NAS *vs*. 75–250 bp for NGS).

Our results showed the low *C*_t_ value of RT-qPCR and high genome coverage in sputum samples than in pharyngeal swabs from both mild (or moderate) and severe (or critical) COVID-19 patients within ≤4w AFS. The results of the two methods revealed consistent trends of the viral load difference between the two types of samples and the two periods. It has been reported that the viral load in pharyngeal swabs was lower than that in sputum samples, and the *C*_t_ value of RT-qPCR in pharyngeal swabs decreased more rapidly than that in sputum samples (Huang et al., 2020). The significant difference revealed by the genome coverage of NAS indicated that this method and the genome coverage data might make a better tool to evaluate the viral load and perform quantitative monitoring of clinical samples. In addition, the detection and quantitative monitoring of sputum might be more sensitive than pharyngeal swabs, which have been reported to be helpful in evaluating disease progression (Yu et al., 2020).

There are several advantages of this study. Firstly, we performed whole-genome amplification of SARS-CoV-2 and sequencing with ONT real-time sequencing technology, which allowed highly sensitive detection in samples for which the RT-qPCR results were false negative. Secondly, as the *C*_t_ value of RT-qPCR could be a relatively accurate parameter for the viral load, which was highly correlated with the copy number of droplet digital PCR (Yu et al., 2020), we analyzed the correlation of the *C*_t_ value with the genome coverage from our data and found consistency in the two values. We evaluated the performance of NAS based on the viral read number and also found a high sensitivity and an acceptable specificity (97.7 and 88.9%, respectively). Thirdly, we analyzed the time-varying characteristics of the sputum samples and pharyngeal swabs. Although the viral load in a clinical sample taken from a specific site may not be representative of the overall viral burden in SARS-CoV-2-infected carriers, pharyngeal swabs collected from patients confirmed to have COVID-19 beyond 4 weeks should be interpreted with caution (Thi et al., 2020). This method would also be valuable in the detection of other critical clinical pathogens, in particular those that cannot be well tested using routine methods, such as virus and fungi.

This study has a few limitations. Firstly, we only collected clinical samples from one hospital in Wuhan during the COVID-19 epidemic. Moreover, the quality of sample collection was limited to discarded samples, and there was about 6 months between RNA extraction and the RT-qPCR or NAS test. These partly explain the several COVID-19 patients showing negative results, with RT-qPCR showing a much worse performance in terms of sensitivity. On one hand, the storage time of the samples could negatively affect the RNA integrity, then causing failure of primer binding or PCR extension. On the other hand, the kits for reverse transcription and amplification were also different during the RT-qPCR detection and generation of the Nanopore library. Therefore, the differences in the sensitivity could also be partially explained by the differences in kits used for reverse transcription and amplification. Furthermore, the numbers of the different types of samples were not balanced; however, it was consistent with clinical practice. In addition, we did not obtain complete genome from any sample. On one hand, the samples were stored for months from the collection to the RNA extraction; therefore the RNA might have degraded partly. On the other hand, the amplification efficiencies of the primers covering the whole viral genome were not equal; therefore, parts of the genomes would not have been amplified and sequenced well.

Here, we evaluated a detection solution based on Oxford nanopore sequencing that showed increased accuracy and sensitivity, as well as saving time, by performing viral genome amplification and nanopore sequencing on SARS-CoV-2 detection. Our results showed the high sensitivity and specificity of this method compared with RT-qPCR. However, the higher costs and longer detection time limited its application at present. It is more suitable as a supplementary method for RT-qPCR now, e.g., while facing the emergence of a new Omicron variant. The sequencing results were also used as an indicator of viral load to display the viral dynamics during infection. This study proved the wide application prospect of nanopore sequencing detection of SAR-CoV-2 and provided more knowledge about the clinical characteristics of COVID-19. This approach would also be suitable for the development of more rapid and sensitive detection methods for critical clinical pathogens to improve the infectious disease diagnostics and disease control measurements.

## Data Availability Statement

The raw sequence data reported in this paper have been deposited in the Genome Sequence Archive (Genomics, Proteomics & Bioinformatics 2021) in National Genomics Data Center (Nucleic Acids Res 2021), China National Center for Bioinformation/Beijing Institute of Genomics, Chinese Academy of Sciences (GSA: CRA004486) that are publicly accessible at https://ngdc.cncb.ac.cn/gsa.

## Ethics Statement

The studies involving human participants were reviewed and approved by the Ethical Review Board of Peking University Third Hospital. The ethics committee waived the requirement of written informed consent for participation.

## Author Contributions

X-XL: conceptualization, methodology, data curation, and writing – original draft. CL: conceptualization, data curation, and writing – editing. P-CD: investigation, formal analysis, visualization, and writing – original draft. Z-QZ, T-TL, and S-YL: investigation. LY: methodology, supervision, and writing – editing. C-KZ: writing – editing. S-CZ: formal analysis. YZ: writing – editing. C-RD: conceptualization, methodology, validation, and supervision. Q-GG: conceptualization, validation, and supervision. All authors took part in the final version for submission and accepted overall accountability for accuracy and integrity of the manuscript.

## Conflict of Interest

P-CD, LY, Z-QZ, T-TL, C-KZ, and S-CZ were employed by Beijing YuanShengKangTai (ProtoDNA) Genetech Co. Ltd., Beijing, China. The remaining authors declare that the research was conducted in the absence of any commercial or financial relationships that could be construed as a potential conflict of interest.

## Publisher’s Note

All claims expressed in this article are solely those of the authors and do not necessarily represent those of their affiliated organizations, or those of the publisher, the editors and the reviewers. Any product that may be evaluated in this article, or claim that may be made by its manufacturer, is not guaranteed or endorsed by the publisher.

## Footnotes

1
www.nhc.gov.cn/yzygj/s7653p/202002/8334a8326dd94d329df351d7da 8aefc2.shtml

2
https://www.protocols.io/view/ncov-2019-sequencing-protocol-bbmuik6w

3
https://github.com/wdecoster/nanofilt

## Abbreviations

AUC: area under the ROC curve
COVID-19: coronavirus disease 2019
*C*_t_: cycle threshold
MAPQ: mapping quality score
NAS: nanopore-based genome amplification and sequencing
NC: negative controls
NGS: next-generation sequencing
AFS: after first showing symptoms
RNA: ribonucleic acid
ROC: receiver operating characteristic
RT-qPCR: reverse transcription quantitative polymerase chain reaction
SARS-CoV-2: severe acute respiratory syndrome coronavirus 2
WHO: world health organization.
